# SOCIOECONOMIC DISPARITIES IN THE MEDICARE HOSPICE CARE INDEX (HCI) INDICATORS

**DOI:** 10.1093/geroni/igad104.1014

**Published:** 2023-12-21

**Authors:** Michael Plotzke, Thomas Christian

**Affiliations:** Abt Associates, St. Louis, Missouri, United States; Abt Associates, Cambridge, Massachusetts, United States

## Abstract

The Centers for Medicare & Medicaid Services’ Hospice Care Index (HCI) measures quality using ten claims-based indicators characterizing hospice service provision. We examine socioeconomic disparities amongst those indicators for hospice users using Medicare claims data from Federal Fiscal Years 2020-2021. We estimate ten ordinary least squares regression models where the dependent variables are each of the ten indicators and the independent variables are provider- and beneficiary-level characteristics. To examine socioeconomic disparities, we also include indicators for beneficiary race/ethnicity, dual eligibility for Medicaid status, urban/rural status, and an area deprivation index. The model with all control variables showed the smallest difference between the socioeconomic variables. For example, the average probability of a Black beneficiary having a least an eight-day gap between nursing visits is 60.3% versus 58.9% (p-value < 0.01) for a white beneficiary. If we only control for socioeconomic variables and no hospice or provider-level controls, we find larger differences, with the average probability of a Black beneficiary having a least an eight-day gap between nursing visits equal to 62.4% versus 58.7% (p-value < 0.01) for a white beneficiary. The results varied by the HCI indicator as well as the socioeconomic variable being examined. These findings suggest that two otherwise similar beneficiaries from different socioeconomic groups are, on average, treated equally by the same hospice. Instead, socioeconomic disparities for some indicators result from how beneficiaries sort into hospices, where the lowest quality hospices are more likely to treat the most disadvantaged.

